# Hepatitis B Virus Infection Alters Gut Microbiota Composition in Mice

**DOI:** 10.3389/fcimb.2019.00377

**Published:** 2019-11-05

**Authors:** Qingfeng Zhu, Panpan Xia, Xin Zhou, Xiaoran Li, Weina Guo, Bin Zhu, Xin Zheng, Baoju Wang, Dongliang Yang, Junzhong Wang

**Affiliations:** Department of Infectious Diseases, Union Hospital, Tongji Medical College, Huazhong University of Science and Technology, Wuhan, China

**Keywords:** HBV infection, HBV mouse model, gut microbiota, microbiota composition, dynamic changes, immune response

## Abstract

Gut microbiota composition is known to be associated with the progression of hepatitis B virus (HBV)-related liver cirrhosis in humans, outcome of HBV infection in mice, and seroconversion of HBV e-antigen in nucleot(s)ide analog-treated patients. The dynamic alteration of the gut microbiota following HBV infection is still unknown. In this study, a hydrodynamic injection mouse model mimicking acute or chronic HBV infection in humans with comparable virological and immunological features was used. The composition of gut microbiota in the control mice and mice with acute or chronic HBV infection was analyzed at different time points using the Illumina MiSeq platform. The expression of immune molecules in the colon was detected by real-time polymerase chain reaction. We found that the changes in gut microbiota composition, including the total operational taxonomic unit (OTU) count and Shannon-Weaver index, were significantly delayed in mice with HBV infection. Furthermore, the ratio of *Bacteroidetes* and *Firmicutes* was stable in the control mice, whereas remarkable dynamic patterns were observed in mice with HBV infection. Interestingly, the dynamic changes in *Lactobacillus* and *Bifidobacterium* were found to differ in acute or chronic HBV infection. In addition, the expression of IFN-γ and PD-L1 in the colon was found to be up-regulated early in mice with acute HBV infection, whereas the expression of PD-L1 in the colon of mice with chronic HBV infection was up-regulated later. These data indicate that HBV infection could hamper the development of the gut microbiota community and dynamically change the gut *Firmicutes*/*Bacteroidetes* ratio. These data improve our understanding of the relationship between gut microbiota and HBV infection.

## Introduction

Approximately a trillion microbial cells colonize the mammalian intestine; these are collectively termed gut microbiota. Gut microbiota play a critical role in several physiological and pathological processes, influencing host immunity and metabolism. The healthy gut microbiota can be characterized by the richness of the gut ecosystem, its amenability to perturbation, and its ability to return to the pre-perturbation state in terms of its diversity, stability, resistance, and resilience. Gut microbiota dysbiosis is described as a compositional and functional alteration in the gut microbiota that is driven by a set of environment- and host-related factors that perturb the microbial ecosystem to an extent that exceeds its resistance and resilience capabilities (Levy et al., 2017). Gut dysbiosis is related to not only intestinal but also extra-intestinal diseases, including nervous system, respiratory system, cardiovascular system, and liver diseases (Lynch and Pedersen, 2016; Gilbert et al., 2018).

The liver is the largest internal organ and gland in the human body, which receives blood from both the portal vein and hepatic artery. The liver is exposed to gut microbes under some disease conditions, including liver cirrhosis (Hackstein et al., 2017), cholestatic liver disease (Tedesco et al., 2018), autoimmune hepatitis (Manfredo Vieira et al., 2018), and intestinal inflammation induced by dextran sodium sulfate (Balmer et al., 2014). Previous studies have showed that live commensal bacteria can be sampled by intestinal dendritic cells (DCs) and transferred to the liver via the lymphatic route or portal vein (Rescigno et al., 2001; Macpherson and Uhr, 2004; Fung et al., 2016). However, the liver can act as a second firewall partially because Kupffer cells can clear commensal bacteria from the systemic vasculature, mirroring their role in pathogen clearance (Balmer et al., 2014).

Hepatitis B virus (HBV) is a human hepadnavirus that causes acute and chronic hepatitis and hepatocellular carcinoma. HBV surface antigen (HBsAg) is commonly regarded as a serological marker for HBV infection. The disappearance of HBsAg and emergence of HBV surface antibody (HBsAb) imply recovery from an infection and the development of immunity against HBV. HBV core antigen (HBcAg) consists of the virus core particle, which is surrounded by HBsAg. HBcAg cannot be detected in the serum, but could be detected in the liver tissue. HBcAg exhibits a strong antigenicity and can induce host to develop core antibodies (HBcAb) rapidly after HBV infection. HBV e antigen (HBeAg), the soluble secretory form of HBcAg, serves as an important serological marker indicating viral replication. The conversion from HBeAg to e antibody (HBeAb) indicates host immune control and low HBV replication (Ganem and Prince, 2004). Gut microbiota dysbiosis has been found in several types of chronic liver diseases including chronic HBV infection, and is associated with disease progression (Tripathi et al., 2018). In patients with hepatitis B-related cirrhosis, the gut microbiota community and metabolism mediated by the gut microbiota were significantly changed when compared with those in healthy controls (Wei et al., 2013). In these patients, the relative abundance of *Bacteroidetes* was found to be negatively correlated with Child–Turcotte–Pugh score, while Enterobacteriaceae members and *Veillonella* showed a positive correlation. The compositional and metabolic changes in the gut microbiota were also found consistently in patients with chronic hepatitis B (Wang et al., 2017). Reconstitution of the gut microbiota using fecal microbiota transplantation facilitated HBeAg clearance in patients with HBeAg-positive chronic hepatitis B after long-term antiviral therapy (Ren et al., 2017). In mice, gut microbiota depletion was found to impair HBV-specific T cell response and prolong HBV infection (Chou et al., 2015). Although prior research has shown that gut microbiota may play a crucial role in HBV infection, the dynamic alterations in gut microbiota following HBV infection is not well-understood.

HBV plasmid hydrodynamic injection (HI) mouse model was established by Yang et al. (2002) and widely used in HBV research (Chou et al., 2015; Ebert et al., 2015). The outcomes of HBV infection in this model depend on the mouse strain and plasmid backbone (Huang et al., 2006). C57BL/6 mice injected with pAAV/HBV1.2 plasmids were found to have persistent HBV infection (Huang et al., 2006), while pSM2/HBV can induce HBV transient infection (Ma et al., 2017). In this study, we investigated the gut microbiota composition at different time points following HBV infection in the HI mouse model with acute or chronic HBV infection.

## Materials and Methods

### Animal Experiments

Male C57BL/6 mice at 5–7 weeks of age were purchased from Hunan SJA Laboratory Animal Co., Ltd. (Hunan, China) and maintained under pathogen-free conditions in the Experimental Animal Centre of Tongji Medical College, Huazhong University of Science and Technology.

All animal experiments were performed in accordance with the guidelines for the Care and Use of Laboratory Animals of the National Institutes of Health, and all the protocols for animal experiments were approved by the Institutional Animal Care and Use Committee at Tongji Medical College, Huazhong University of Science and Technology (Permit Number: S814).

Two plasmids, pSM2/HBV (provided by Dr. Hans Will, Heinrich-Pette-Institute, Hamburg, Germany) and pAAV/HBV1.2 (provided by Prof. Chen PJ, Graduate Institute of Clinical Medicine, College of Medicine, National Taiwan University), were used in this study. Mice at 6–8 weeks of age (after 1 week of acclimatization) were hydrodynamically injected with HBV plasmid DNA as described in previous studies (Huang et al., 2006; Wang et al., 2014b). Briefly, 10 μg of HBV plasmids was diluted with phosphate-buffered saline (PBS) equivalent to 0.1 mL/g of the mouse body weight, and the total volume of HBV plasmid DNA was injected into the tail vein of mice within 5–8 s. The control mice were hydrodynamically injected with PBS. The mice were observed for 11 weeks after HI.

### Detection of HBsAg, HBsAb, HBcAb, and HBV DNA in the Serum, and HBcAg in the Liver Tissue of Mice

The serum of mice was collected and diluted 1:10 with PBS. HBsAg, HBsAb, HBeAg, HBeAb, and HBcAb were detected using an ELISA kit (Kehua Bio-engineering Co. Ltd., Shanghai, China), per the manufacturer's instructions. The viral load was quantified by real-time polymerase chain reaction (PCR) using SYBR Green dye (Sigma-Aldrich, St. Louis, MO, USA) as described previously (Wang et al., 2014b). HBcAg in the liver tissue was detected by immunohistochemistry. The liver tissue was collected, embedded in paraffin, and sectioned. The sections were stained with rabbit anti- HBcAg polyclonal antibody (Dako, Japan) and visualized using the DAKO EnVision™ Detection Systems (Dako, Japan), according to the manufacturer's instructions.

### Fecal Sample Collection and DNA Amplification

Fecal samples from the mice were collected and immediately frozen at −80°C. Bacterial DNA in the fecal samples was extracted using the E. Z. N. A. soil DNA Kit (Omega Bio-tek, Norcross, GA, USA). The V3–V4 hypervariable regions of the bacterial 16S rRNA gene were amplified with primers 338F (5′-ACTCCTACGGGAGGCAGCAG-3′) and 806R (5′-GGACTACHVGGGTWTCTAAT-3′) using a thermocycler PCR system (GeneAmp 9700; ABI, Carlsbad, CA, USA). The PCRs were run under the following conditions: 95°C for 3 min (30 s at 95°C, 30 s at 55°C, and 45 s at 72°C) for 27 cycles, and 10 min at 72°C. The resulting PCR products were extracted from a 2% agarose gel and further purified using the AxyPrep DNA Gel Extraction Kit (Axygen Biosciences, Union City, CA, USA) and quantified using QuantiFluor™-ST (Promega, Madison, WI, USA).

### 16S rRNA Sequencing and Processing of Sequenced Data

Purified amplicons were pooled in equimolar ratios and paired-end sequenced (2 × 300) on an Illumina MiSeq platform (Illumina, San Diego, USA) following the standard protocols as previous reported (Lin et al., 2018) by Majorbio Bio-Pharm Technology Co. Ltd. (Shanghai, China). The sequencing data have been deposited in the NCBI Sequence Read Archive (SRA) database (Accession Number: SRP174629).

Raw fastq files were demultiplexed, quality-filtered using Trimmomatic and merged by FLASH using the following criteria: (i) the reads were truncated at any site receiving an average quality score of <20 over a 50-bp sliding window; (ii) primers were matched allowing two nucleotide mismatching, and reads containing ambiguous bases were removed; and (iii) sequences whose overlap exceeded 10 bp were merged according to their overlapping sequence.

Operational taxonomic units (OTUs) were clustered with a 97% similarity cutoff using UPARSE (version 7.1 http://drive5.com/uparse/), and chimeric sequences were identified and removed using UCHIME. The taxonomy of each 16S rRNA gene sequence was assigned using RDP Classifier algorithm (http://rdp.cme.msu.edu/) against the Silva (SSU128) 16S rRNA database using a confidence threshold of 70%. Further details of bioinformatics analysis are provided in the Supplementary Material.

### Cellular Isolation and Flow Cytometry

Lymphocytes in the liver were isolated as described in a previous study (Ma et al., 2017). Briefly, the mouse liver was perfused with PBS and then digested with an enzyme solution containing 0.05% collagenase type IV (Sigma-Aldrich), 0.002% DNAase I (Sigma-Aldrich), and 10% fetal bovine serum for 30 min. Lymphocytes in the homogenate were isolated using Percoll (Sigma-Aldrich), following the manufacturer's instructions and cultured in RPMI 1640 medium in 96-well plates and stimulated with CD8+ T cell epitope (K^b^-HBV Cor_93−100_ epitope, MGLKFRQL, 10 μg/mL). For cell surface staining, the cells were stained with BV421-anti-CD8 (eBioscience, San Diego, CA, USA). For intracellular cytokine staining, the cells were fixed and permeabilized using the Intracellular Fixation and Permeabilization Buffer Set (Invitrogen, Carlsbad, CA), and then stained with the following antibodies: APC-anti-IFN-γ, PE-anti-IL-2, and FITC-anti-TNF-α (Biolegend, San Diego, CA, USA). All the samples were stained with Fixable Viability Dye eFluor 506 (eBioscience) to exclude dead cells. The stained cells were analyzed using a BD FACSCanto II flow cytometer. Data were analyzed using FlowJo software (Tree Star, Ashland, OR, USA).

### Cytokine Expression in the Colon

The relative mRNA levels of cytokines in the colon samples were measured by reverse-transcription (RT)-qPCR. Briefly, the colon tissue from the control, pSM2/HBV HI, and pAAV/HBV1.2 HI mice were collected after euthanizing the mice. The samples were snap-frozen in liquid nitrogen and stored at −80°C for not more than 3 months until RNA extraction. The total RNA was extracted from tissue samples using TRIzol reagent (Invitrogen, Carlsbad, CA, USA) according to the manufacturer's instruction. The quality and quantity of the RNA sample were evaluated by calculating the A_260_/A_280_ ratio and 28S/18S ribosomal RNA ratio. The A_260_/A_280_ ratio ranged from 1.90 to 2.10 and the 28S/18S ribosomal RNA ratio ranged from 1.8 to 2.0. RT-qPCR was conducted using the RNA templates and SYBR Green PCR Kit (Qiagen, Dusseldorf, NRW, Germany) according to the manufacturer's instructions on the CFX Connect™ Real-Time PCR Detection System (BIO-RAD, Hercules, California, USA). RT-qPCR was carried out in a reaction mixture of volume 20 μL, containing 10 μL of 2 × Buffer (dNTP mixture, Mg^2+^ and SYBR Green I), 0.8 μL of enzyme mix (RTase, RNase inhibitor, and Ex Taq), 0.8 μL of 10 μM forward primers, 0.8 μL of 10 μM reverse primers, 2 μL of 50 ng/μL total RNA, and 5.6 μL of RNase free dH_2_O. The cycling conditions were as follows: 42°C for 5 min, 95°C for 10 s, followed by 40 cycles of 5 s at 95°C and 30 s at 60°C. QuantiTect Primers (Qiagen, Dusseldorf, NRW, Germany) were used according to the manufacturer's instructions. The primer specificity was validated using the melting curve analysis. The primer efficiency was evaluated using the calibration curve generated with serial dilutions (covering three orders of magnitude) of cDNA. The efficiency of all primer pairs ranged from 0.92 to 1.08. The *R*^2^ values (correlation coefficients) were between 0.992 and 0.997. No-template controls were included to ensure the absence of reagent contamination and genomic DNA. To draw the melting curve, the PCR products were heated from 65 to 95°C (0.5°C/5 s), and the raw Ct values were obtained. All of the reactions were performed in triplicate. The slope of the standard curve generated through serial 10-fold dilutions of the PCR products was used to calculate the amplification efficiency of each candidate gene. Beta-actin expression was used to normalize the mRNA expression levels of each candidate gene, and the expression levels are presented as the copy number/10^5^ copies of β*-actin* mRNA.

### Statistical Analysis

Statistical analyses were performed using SPSS software version 12.0 (SPSS Inc, Chicago, IL, USA). Two-tailed unpaired Student's *t*-tests and one-way ANOVA with Tukey's multiple comparison tests were used to analyze the differences between two groups and among multiple groups, respectively. The results with a *p* < 0.05 were considered statistically significant.

## Results

### Transient and Persistent HBV Infection in Mice Following Hydrodynamic Injection (HI) of pSM2/HBV or pAAV/HBV1.2 Plasmid

**Figure 1:**
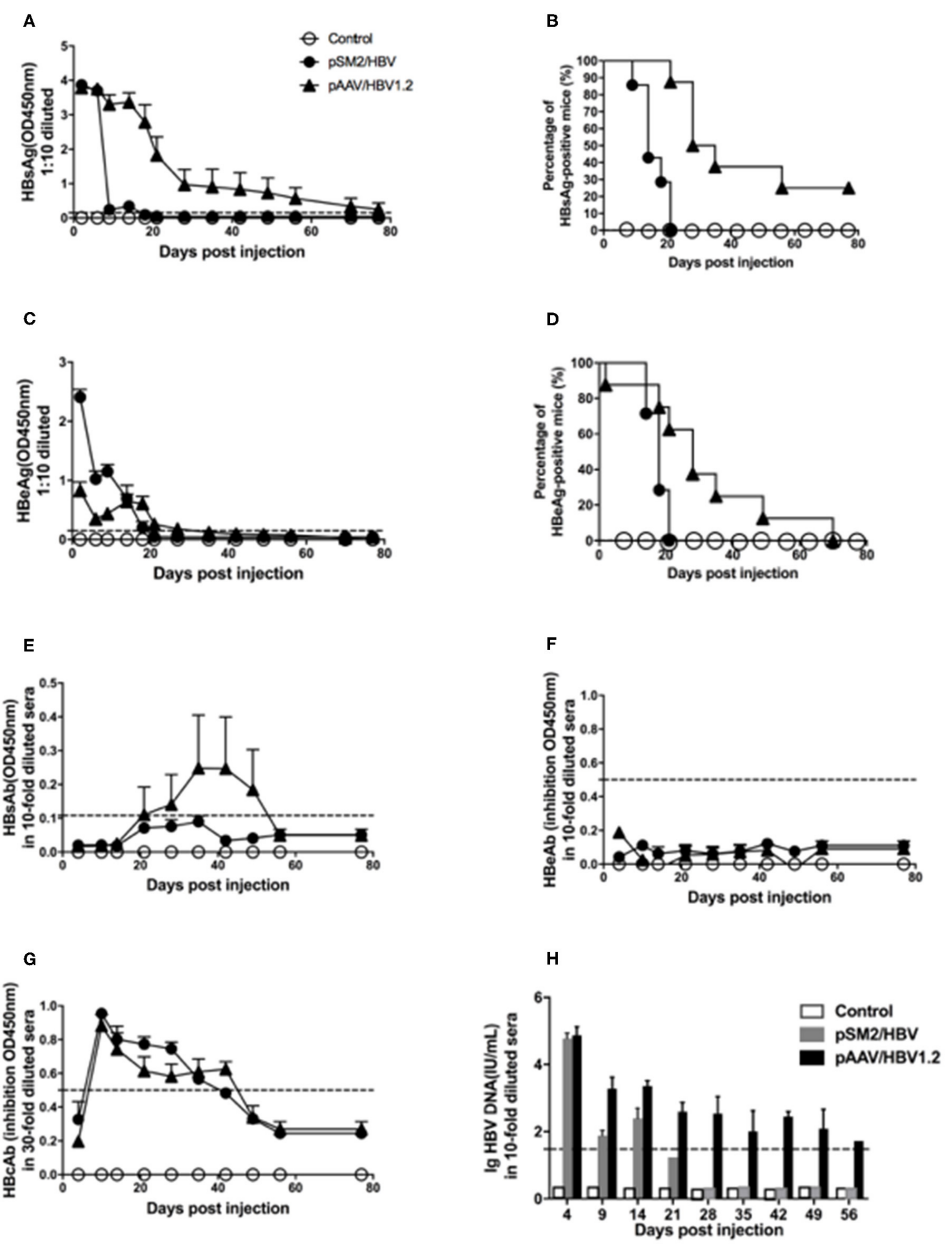
Dynamic changes in HBV antigens, HBV antibodies, and HBV DNA in the serum of mice hydrodynamically injected (HI) with pSM2/HBV or pAAV/HBV1.2. Mice were HI with pSM2/HBV or pAAV/HBV1.2 plasmids. The control mice were HI with PBS. HBsAg **(A)**, HBeAg **(C)**, HBsAb **(E)**, HBeAb **(F)**, and HBcAb **(G)** in the serum at different time points were detected using the ELISA. The positive rate of HBsAg **(B)** and HBeAg **(D)** in the serum at different time points is shown as Kaplan–Meier curves. Serum HBV DNA **(H)** was detected using real-time PCR. The cutoff values are shown as dotted lines. Control mice, *n* = 10; pSM2/HBV HI mice, *n* = 9; pAAV/HBV1.2 HI mice, *n* = 9.

Consistent with the findings of a previous study, we found that HBsAg, HBeAg, and HBV DNA were detected 1 day after HI, decreased rapidly, and cleared within 21 days after HI of the pSM2/HBV plasmid in mice. While in mice injected with the pAAV/HBV1.2 plasmid, HBsAg, HBeAg, and HBV DNA were also detected 1 day after HI, then decreased gradually, and persisted for at least 56 days after HI. HBsAb was detected shortly after HBsAg clearance in mice injected with the pSM2/HBV plasmid, while it was consistently undetected in those injected with the pAAV/HBV1.2 plasmid. HBcAb was detected, while HBeAb was not, in both groups. In the control mice, all the above serological markers were negative during the whole observation period, as expected (Figure 1). HBcAg in the liver was examined by immunohistochemistry (Supplementary Figure S1). Consistent with HBsAg and HBV DNA in the peripheral blood, HBcAg could be detected on day 14 in pSM2/HBV HI mice, while could be detected both on days 14 and 49 in pAAV/HBV1.2 HI mice. HE staining indicated that no inflammation was induced after HI of HBV plasmids (data not shown), which was consistent with the findings of a previous study. There was no significant difference in body weight among the control, pSM2/HBV HI, and pAAV/HBV1.2 HI mice (Supplementary Figure S2).

**Figure 2:**
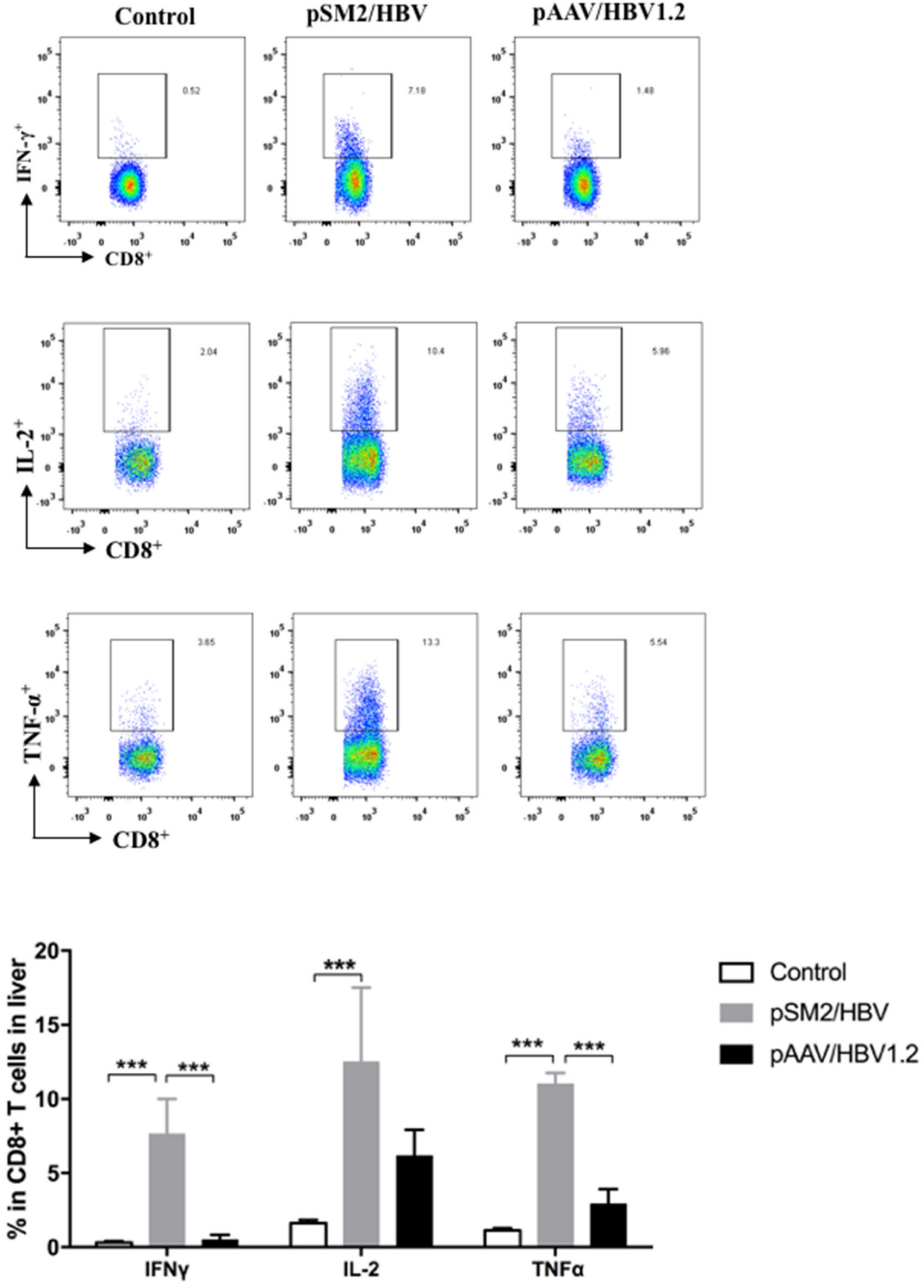
HBV specific CD8^+^ T cell responses in the control, pSM2/HBV HI, and pAAV/HBV1.2 HI mice. On day 14 after HI, liver infiltrated lymphocytes were isolated and stimulated with HBV core peptide. The frequency of CD8^+^IFN-γ^+^, CD8^+^IL-2^+^, and CD8^+^TNF-α^+^ cells was detected using FACS. Control mice, *n* = 7; pSM2/HBV HI mice, *n* = 5; pAAV/HBV1.2 HI mice, *n* = 5. ***p < 0.001.

The viral-specific T cell response is the key factor that determines the outcome of HBV infection, especially during the early phase of infection. Therefore, the viral specific T cell response in the liver was analyzed by FACS on day 14 after HI. We found that significantly higher frequency of CD8+ T cells expressing IFN-γ, TNF-α, and IL-2 were detected in the pSM2/HBV group than that in the control and pAAV/HBV1.2 groups (Figure 2). The findings suggest that both the virological and immunological features of pSM2/HBV HI and pAAV/HBV1.2 HI mice mimic acute (transient viral replication and robust immune response) and chronic (persistent viral response and weak immune response) HBV infections, respectively.

### HBV Infection Delays the Development of the Gut Microbiota

**Figure 3:**
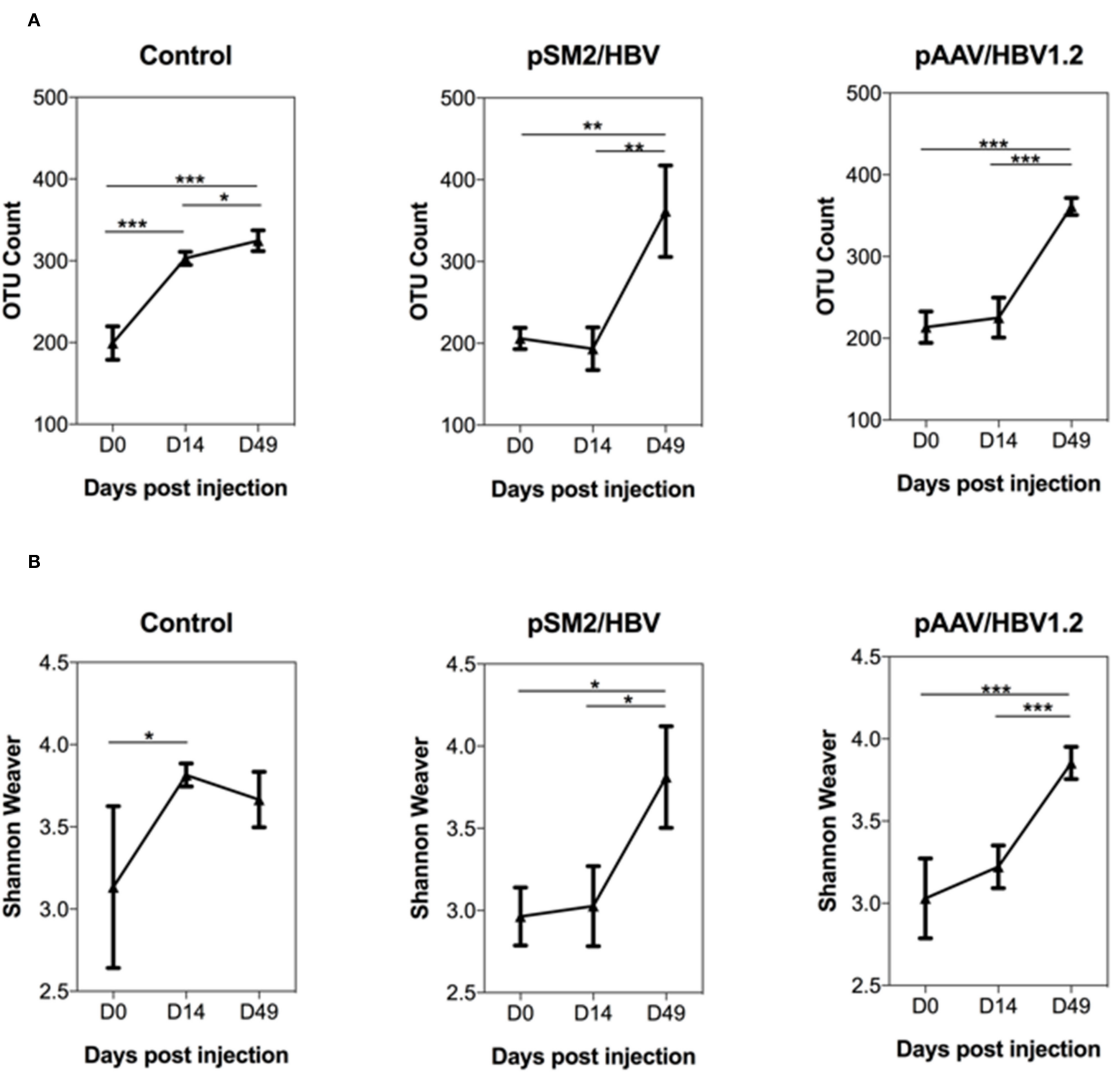
Dynamic changes in the overall abundance and diversity of gut microbiota in the control, pSM2/HBV HI, and pAAV/HBV1.2 HI mice. The OTU count **(A)** and Shannon–Weaver index **(B)** of the gut microbiota at different time points in the control, pSM2/HBV HI, and pAAV/HBV1.2 HI mice are shown. Control mice, *n* = 3; pSM2/HBV HI mice, *n* = 3; pAAV/HBV1.2 HI mice, *n* = 3–7. *p < 0.05, **p < 0.01, ***p < 0.001.

In this study, 16S rRNA sequencing was used to evaluate the gut microbiome community. A total of 39 23 860 reads were generated, and 19 61 930 sequences remained after quality filtering. The average sequencing depth was 53 025 per sample. The rarefaction curves illustrated that the bacterial OTUs obtained by the current sequencing depth were sufficient to represent the microbial communities (Supplementary Figure S3). First, we analyzed the dynamic changes in the total observed bacterial OTU numbers in the feces of control mice and mice with acute or chronic HBV infection. We found that the gut microbiota OTU number before HI was comparable in all the three groups of mice. In the control mice, the gut microbiota OTU number was significantly increased by days 14 and 49 after HI. However, in mice HI with pSM2/HBV or pAAV/HBV1.2, the gut microbiota OTU number remained at comparable levels on days 0 and 14 after HI, but increased significantly by day 49 after HI (Figure 3A).

The dynamic changes in gut microbiota diversity were then analyzed using the Shannon-Weaver index, and similar results were obtained. The diversity of gut microbiota increased significantly by day 14 after HI in the control mice, while this increase was not observed until day 49 after HI in mice injected with pSM2/HBV or pAAV/HBV1.2 (Figure 3B). Faith's phylogenetic diversity and beta diversity PCoA plots were also constructed. In the control mice, Faith's phylogenetic diversity was increased significantly on day 14 after HI, but in pSM2/HBV and pAAV/HBV1.2 HI mice, it was increased significantly until day 49 after HI, which is similar with that of the Shannon–Weaver index (Supplementary Figure S4). The PCoA of OTU levels indicated that the beta diversity changed in all the three groups, although the results were not consistent with each other in weighted unifrac, unweighted unifrac, and Bary-Curtis distances. It also did not exactly match the OTU number, Shannon–Weaver index, and Faith's phylogenetic diversity, which may be due to the small sample used in the study (Supplementary Figure S5). These results indicated that the gut microbiota community in C57BL/6 mice develops between 8 and 10 weeks of age. In contrast, this development of mice gut microbiota was hampered by either transient or persistent HBV infection.

### HBV Infection Alters the Dynamic Changes in Gut Microbiota

**Figure 4:**
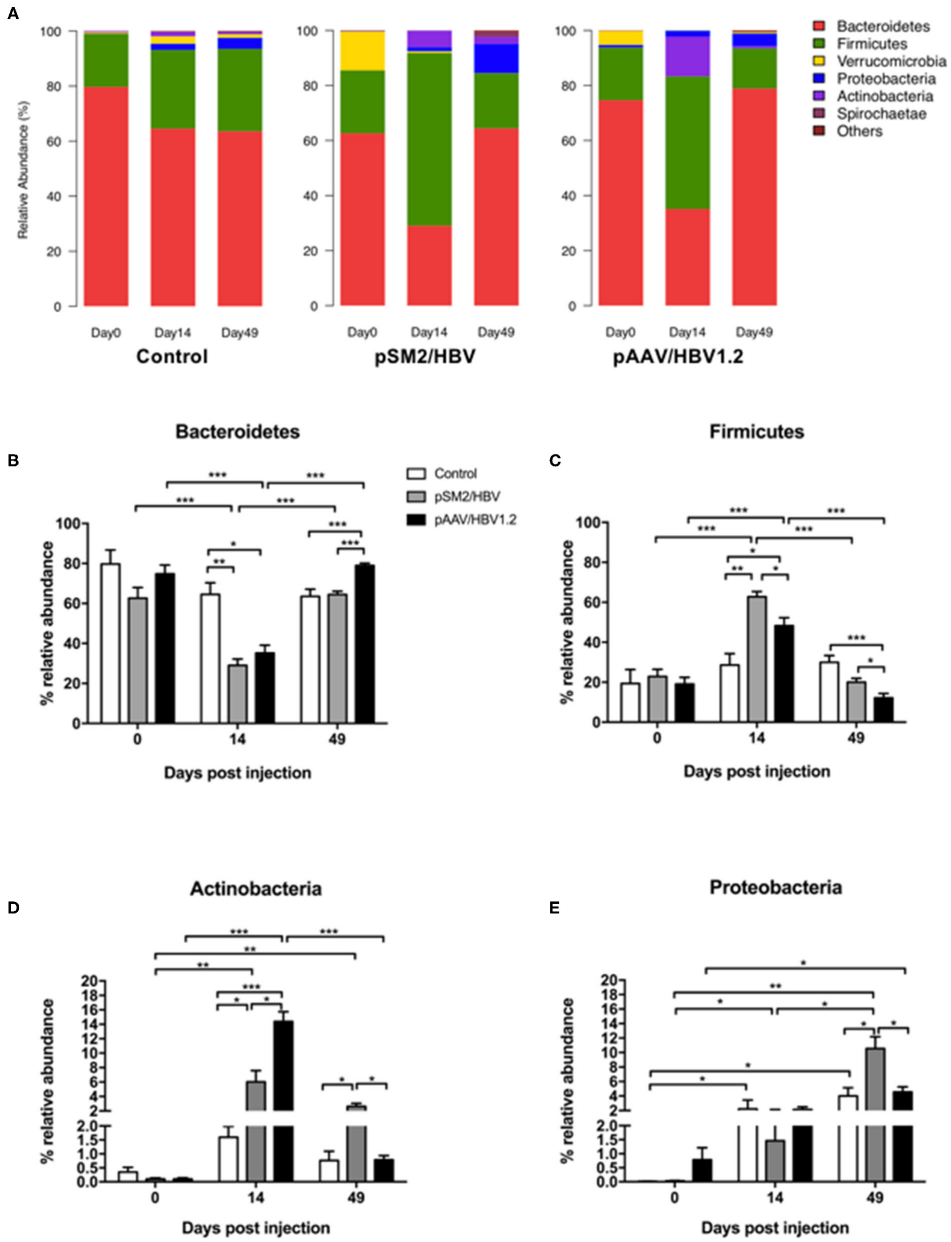
Phylum level dynamic changes in gut microbiota in the control, pSM2/HBV HI, and pAAV/HBV1.2 HI mice. The phylum-level composition of gut microbiota **(A)** and the relative abundance of Bacteroidetes **(B)**, Firmicutes **(C)**, Proteobacteria **(D)**, and Actinobacteria **(E)** in the control, pSM2/HBV HI, and pAAV/HBV1.2 HI mice at different time points. Control mice, *n* = 3; pSM2/HBV HI mice, *n* = 3; pAAV/HBV1.2 HI mice, *n* = 3–7. *p < 0.05, **p < 0.01, ***p < 0.001.

We investigated the dynamic changes of the gut microbiota population in the control, pSM2/HBV HI, and pAAV/HBV1.2 HI mice at the phylum, family, and genus levels. We found that the gut bacterial distribution in the mice was dominated by six phyla, namely, *Bacteroidetes, Firmicutes, Verrucomicrobia, Proteobacteria, Actinobacteria*, and *Spirochaetes* (Figure 4A).

More than 80% of the gut microbiota was composed of *Bacteroidetes* and *Firmicutes* in the control, pSM2/HBV HI, and pAAV/HBV1.2 HI mice at all time points evaluated. The pattern of bacterial distribution was comparable in the control mice on days 0, 14, and 49, although the abundance of *Proteobacteria, Verrucomicrobia*, and *Actinobacteria* increased by days 14 and 49 when compared with that on day 0 (Figure 4A). In pSM2/HBV and pAAV/HBV1.2 HI mice, the pattern of bacterial distribution changed obviously by day 14 after HI, with a decrease in *Bacteroidetes* and an increase in *Firmicutes* (Figure 4A).

The abundance of each dominant bacterial phylum was analyzed in detail. In the control mice, the abundance of *Bacteroidetes* and *Firmicutes* was stable at all the three time points. However, in both pSM2/HBV and pAAV/HBV1.2 HI mice, the abundance of *Bacteroidetes* decreased significantly by day 14, and then rebounded by day 49. Interestingly, the rebound level in pAAV/HBV1.2 HI mice was higher than that in the control and pSM2/HBV HI mice (Figure 4B). On the contrary, in both pSM2/HBV and pAAV/HBV1.2 HI mice, the abundance of Firmicutes increased significantly by day 14, and then decreased to the baseline level by day 49. The abundance of Firmicutes in pSM2/HBV HI mice was higher than that in pAAV/HBV1.2 HI mice on both days 14 and 49 after HI (Figure 4C). The ratio of *Firmicutes/Bacteroides* increased on day 14 and decreased on day 49 significantly in both pSM2/HBV and pAAV/HBV1.2 HI mice, but it remained at levels comparable with those of the control group. Otherwise, the ratio of *Firmicutes*/*Bacteroides* was higher in pSM2/HBV HI mice than in pAAV/HBV1.2 HI mice on both days 14 and 49 after HI (Supplementary Figure S6).

The dynamic changes observed for *Actinobacteria* and *Proteobacteria* demonstrated a different pattern. The abundance of *Actinobacteria* remained nearly unchanged in the control mice, while it increased significantly by day 14 in both pSM2/HBV and pAAV/HBV1.2 HI mice, with a higher level observed in pAAV/HBV1.2 HI mice. The abundance of *Actinobacteria* then decreased to the baseline level by day 49 in pAAV/HBV1.2 HI mice, but remained elevated in pSM2/HBVHI mice (Figure 4D). The abundance of *Proteobacteria* increased significantly by day 14 and then decreased to the baseline level by day 49 in the control and pAAV/HBV1.2 HI mice, while it gradually increased to the highest level by day 49 in pSM2/HBV HI mice (Figure 4E).

**Figure 5:**
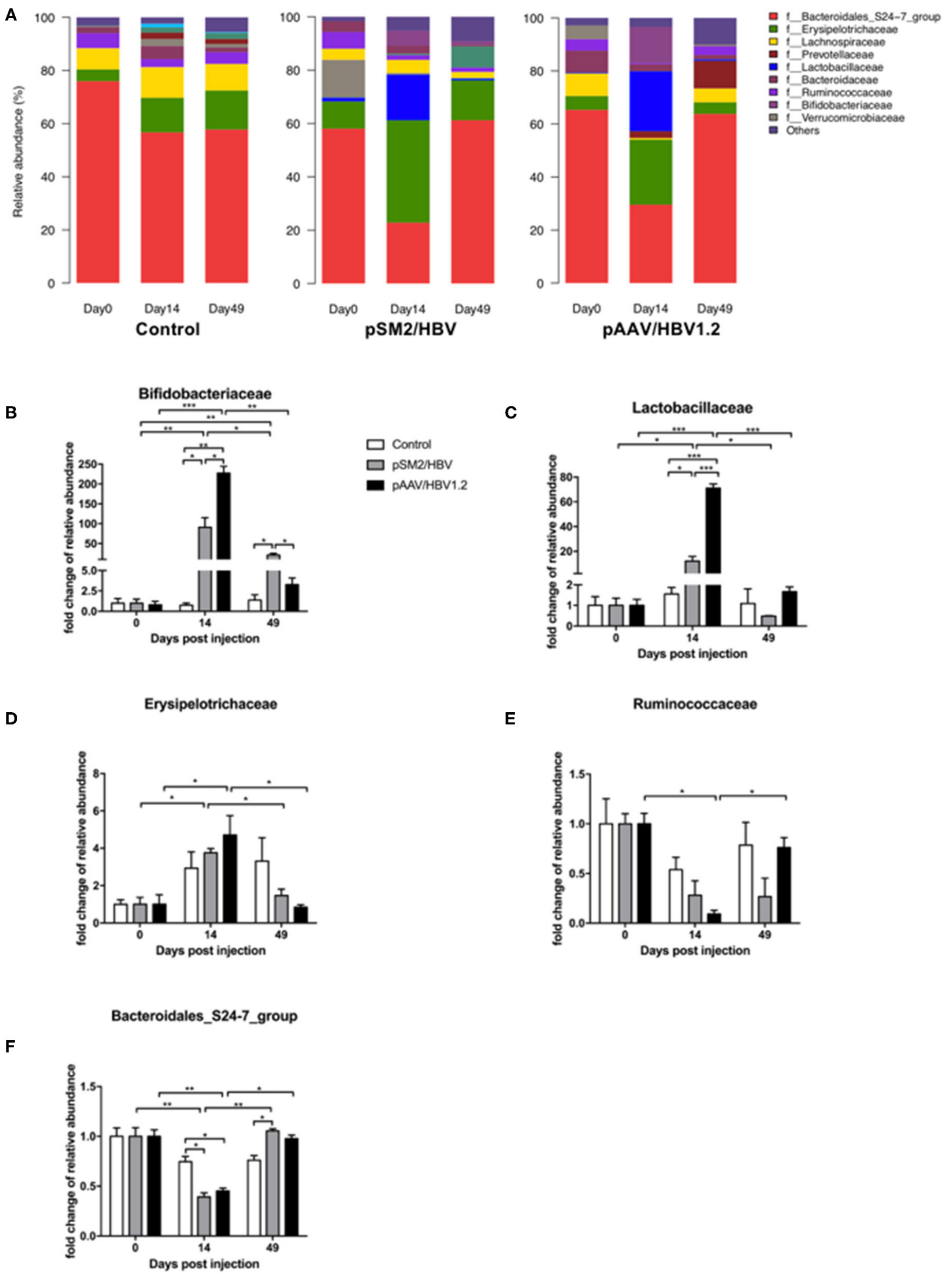
Family-level dynamic changes in gut microbiota in the control, pSM2/HBV HI, and pAAV/HBV1.2 HI mice. Family-level composition of gut microbiota **(A)** and fold changes in the relative abundance of *Bifidobacteriaceae* **(B)**, *Lactobacillaceae* **(C)**, *Erysipelotrichaceae* **(D)**, *Ruminococcaceae* **(E)**, and *Bacteroidales*_S24-7_group **(F)** in the control, pSM2/HBV HI, and pAAV/HBV1.2 HI mice at different time points. The average relative abundance of each bacterium on day 0 was regarded as the baseline. The fold change in the relative abundance of each bacterium at each time point was calculated relative to that baseline. Control mice, *n* = 3; pSM2/HBV HI mice, *n* = 3; pAAV/HBV1.2 HI mice, *n* = 3–7. *p < 0.05, **p < 0.01, ***p < 0.001.

Gut bacterial distribution at the family level was also analyzed (Figure 5A), and five families were found to be related to the outcomes of HBV infection in mice, including *Bifidobacteriaceae* (Figure 5B), *Lactobacillaceae* (Figure 5C), *Erysipelotrichaceae* (Figure 5D), *Ruminococcaceae* (Figure 5E), and *Bacteroidales_S24-7_group* (Figure 5F). *Bifidobacteriaceae*, a family of *Actinobacteria*, demonstrated a similar dynamic pattern as that observed for *Actinobacteria* as a whole. Similarly, *Bacteroidales_S24-7_group*, a member of *Bacteroidetes*, showed a dynamic changing pattern similar to that of *Bacteroidetes*. However, *Lactobacillaceae, Erysipelotrichaceae*, and *Ruminococcaceae*, members of *Firmicutes*, were found to have distinct dynamic changes. The abundance of *Lactobacillaceae* was nearly unchanged in the control mice but increased significantly by day 14 in both pSM2/HBV and pAAV/HBV1.2 HI mice, with a significantly higher level in pAAV/HBV1.2 HI mice. This level then decreased to the baseline by day 49. The abundance of *Erysipelotrichaceae* increased significantly by day 14 and then decreased to the baseline level on day 49 in both pSM2/HBV and pAAV/HBV1.2 HI mice. The abundance of *Ruminococcaceae* decreased significantly by day 14 and then rebounded to the baseline level on day 49 only in pAAV/HBV1.2 HI mice.

**Figure 6:**
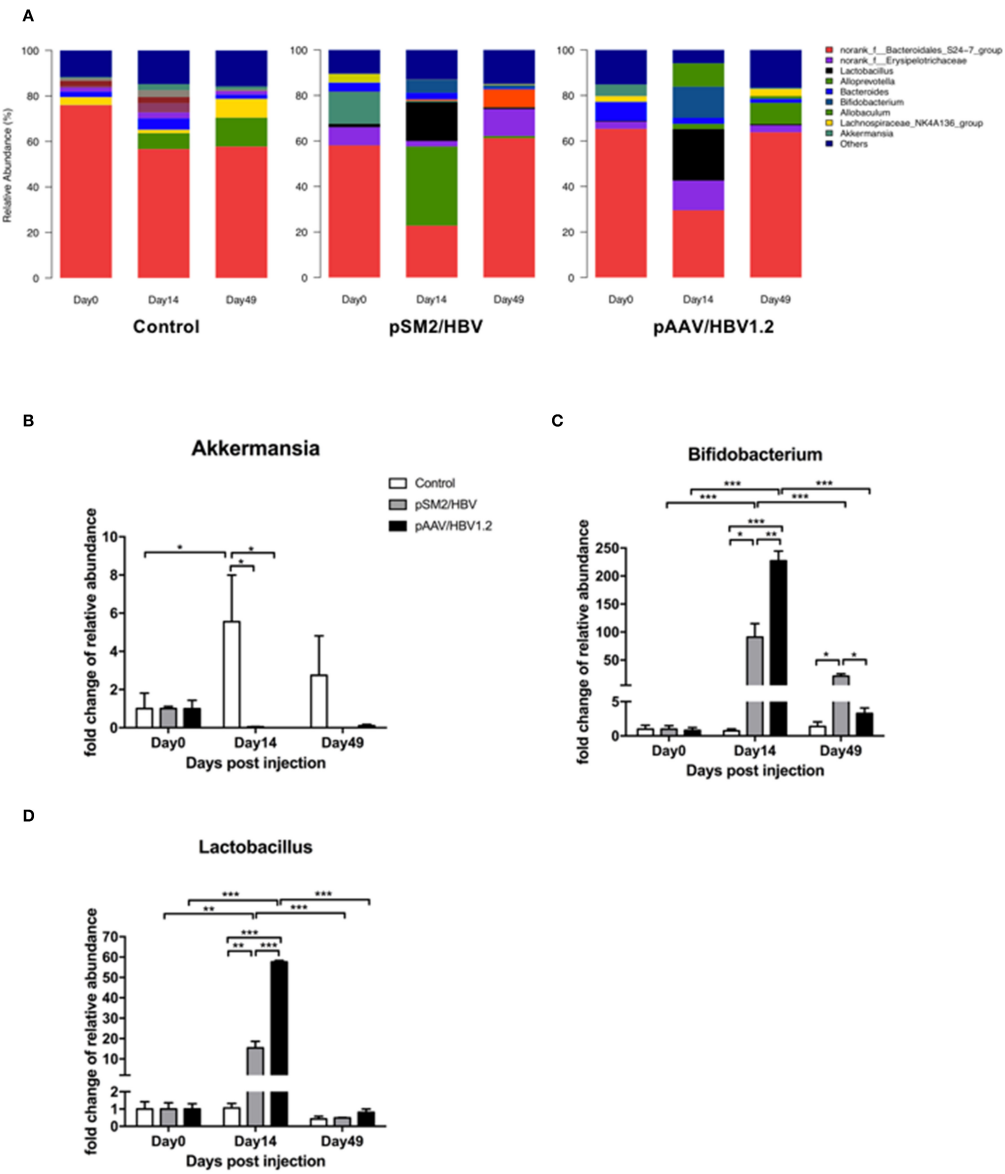
Genus-level dynamic changes in gut microbiota in the control, pSM2/HBV HI, and pAAV/HBV1.2 HI mice. Genus-level composition of gut microbiota **(A)** and fold changes in the relative abundance of *Akkermansia* **(B)**, *Bifidobacterium* **(C)**, and *Lactobacillus* **(D)** in the control, pSM2/HBV HI, and pAAV/HBV1.2 HI mice at different time points. The average relative abundance of each bacterium on day 0 was regarded as the baseline. The fold change in the relative abundance of each bacterium at each time point was calculated relative to that baseline. Control mice, *n* = 3; pSM2/HBV HI mice, *n* = 3; pAAV/HBV1.2 HI mice, *n* = 3–7. *p < 0.05, **p < 0.01, ***p < 0.001.

At the genus level, the dominant bacteria in the gut microbiota included *norank_f_Bacteroidales_S24-7_group, norank_f_Erysipelotrichaceae, Lactobacillus, Alloprevotella, Bacteroides, Bifidobacterium, Allobaculum, Lachnospiraceae_NK4A136_group*, and *Akkermansia*. The pattern of genus-level bacterial distribution was generally comparable in the control mice on days 0, 14, and 49, with the exception of *norank_f_Bacteroidales_S24-7_group*, which decreased by days 14 and 49 when compared with that on day 0. In pSM2/HBV and pAAV/HBV1.2 HI mice, the pattern of bacterial distribution was changed significantly by day 14 after HI (Figure 6A). Furthermore, significant changes were found in the proportion of *Akkermansia* (Figure 6B), *Bifidobacterium* (Figure 6C), and *Lactobacillus* (Figure 6D). *Bifidobacterium*, a member of Bifidobacteriaceae, demonstrated a dynamic changing pattern approximating that of Bifidobacteriaceae as a whole. Similarly, *Lactobacillus*, a member of *Lactobacillaceae*, showed a comparable dynamic changing pattern to that of *Lactobacillaceae*. Although the proportion of *Akkermansia* increased significantly by day 14 in the control mice, it remained nearly unchanged in the pSM2/HBV and pAAV/HBV1.2 HI mice (Figure 6B).

**Figure 7:**
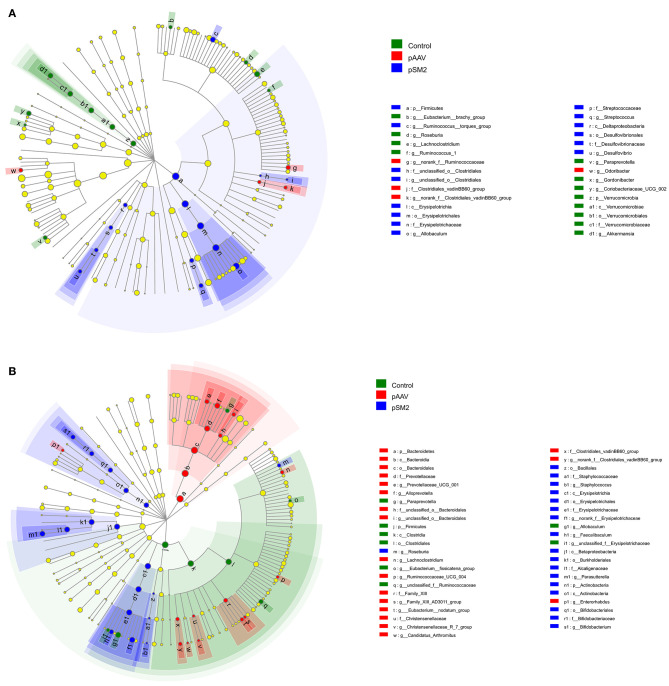
Differences in bacterial richness in the control, pSM2/HBV HI, and pAAV/HBV1.2 HI mice. LEfSe was performed to determine the difference in bacterial richness; the threshold of LDA score was 2.0. The cladogram represents the phylogenetic relationship of significant OTUs associated with each group on days 14 **(A)** and 49 **(B)** after HI.

Furthermore, we identified the OTUs at different taxonomic levels using linear discriminant analysis effect size (LEfSe) among the control, pSM2/HBV, and pAAV/HBV1.2 HI mice compared with the gut microbiota composition at day 0. We found that the gut microbiota composition had changed remarkably on day 14 (but not on day 49) in the control mice, while such changes were observed on both days 14 and 49 after HI in pSM2/HBV and pAAV/HBV1.2 HI mice. On day 14 after HI, 12 OTUs were enriched in the control mice, while only 14 OTUs were enriched in pSM2/HBV HI mice and 4 OTUs were enriched in pAAV/HBV1.2 HI mice (Figure 7A). On day 49 after HI, only eight OTUs were enriched in the control mice, while 18 OTUs were enriched in pSM2/HBV HI mice and 19 OTUs were enriched in pAAV/HBV1.2 HI mice (Figure 7B).

### Histological and Immunological Changes in the Colon

**Figure 8:**
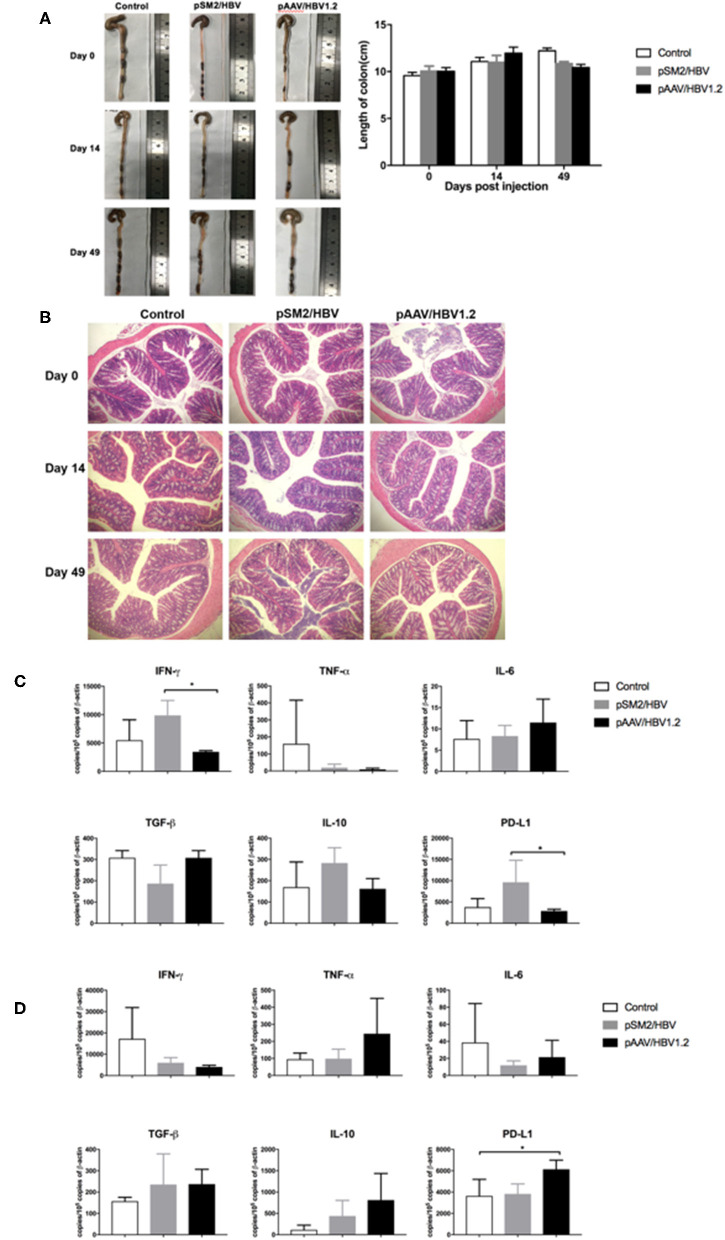
Pathological and immunological changes in the colon of the control, pSM2/HBV HI, and pAAV/HBV1.2 HI mice. **(A)** Macropathologic changes in the colon and colon length, and **(B)** histological changes (HE staining, magnification 200×) in the colon of the control, pSM2/HBV HI, and pAAV/HBV1.2 HI mice at different time points. The mRNA expression levels of immune-related molecules in the colon on days 14 **(C)** and 49 **(D)** after HI as detected by real-time PCR. Control mice, *n* = 3; pSM2/HBV HI mice, *n* = 3; pAAV/HBV1.2 HI mice, *n* = 3–7. *p < 0.05.

To investigate the histological and immunological changes in the colon, we measured the length and immune molecule expression in the colon, as well as performed HE staining. No significant difference was observed in the length of the colon among the control, pSM2/HBV HI, and pAAV/HBV1.2 HI mice on days 0, 14, and 49 after HI (Figure 8A). HE staining showed that the structure of the colon was complete and that no obvious damage was observed in the control, pSM2/HBV HI, and pAAV/HBV1.2 HI mice on days 0, 14, and 49 after HI (Figure 8B).

The expression of immune molecules in the colon was detected by real-time PCR. On day 14 after HI, the expression of IFN-γ and PD-L1 was significantly higher in pSM2/HBV HI mice than in pAAV/HBV1.2 HI mice (Figure 8C). However, on day 49 after HI, the level of PD-L1 was significantly higher in pAAV/HBV1.2 HI mice than in the control and pSM2/HBV HI mice (Figure 8D). No significant difference was found in the expression of TNF-α, IL-6, IL-10, and TGF-β in the colon at the two time points among the three groups.

## Discussion

While recent studies have indicated that gut microbiota may play a crucial role in the course of HBV infection, the dynamic alterations in gut microbiota in acute and chronic HBV infections are not well-known. Using the HBV HI mouse model mimicking acute or chronic HBV infection in humans, we found that the gut microbiota community in the control mice changed remarkably by day 14, based on the total observed OTU counts and Shannon-Weaver index, while this was delayed in mice with HBV infection. We identified six phyla, five families, and nine genera predominant in the mouse gut microbiota, and only one phylum (*Proteobacteria*) and one genus (*Akkermansia*) demonstrated dynamic changes in the control mice, whereas two phyla (*Bacteroidetes* and *Firmicutes*) and two families (*Erysipelotrichaceae* and *Bacteroidales)* demonstrated comparable dynamic changes in mice with HBV infection. The dynamic changes in *Bifidobacterium*, its phylum *Actinobacteria*, and its family *Bifidobacteriaceae* were similar, although the pattern differed in mice with acute or chronic HBV infection compared with that in the control mice. The dynamic changes in *Lactobacillus* and its family *Lactobacillaceae* were also consistent; however, the abundance on day 14 differed in mice with acute or chronic HBV infection. *Ruminococcaceae* showed dynamic changes only in mice with chronic HBV infection. Although there were obvious dynamic alterations in the gut microbiota during acute and chronic HBV infection in mice, there were no histological changes observed in the colon. However, the expression of IFN-γ and PD-L1 in the colon was up-regulated in mice with acute HBV infection relative to chronic HBV infection on day 14, and the expression of PD-L1 in the colon was up-regulated in mice with chronic HBV infection relative to that in the control group on day 49.

Gut microbiota can develop rapidly after birth in both mice and humans. It has been reported that the gut microbiota is established and matured during the first two postnatal years in humans (Planer et al., 2016). Gut microbiota in mice was found to reach a high degree of stability within 1–2 weeks of weaning (Schloss et al., 2012), while was found to establish and mature between birth and 11 weeks of age (Chou et al., 2015). In this study, bacterial richness and gut microbiota diversity (not only Shannon–Weaver index but also Faith's phylogenetic diversity) increased significantly by 14 days after HI (at the age of 8–10 weeks) in the control mice, and stabilized on day 49. This suggested the commensal bacteria that colonized the gut of mice was increasing at the age of 8–10 weeks, which was consistent with the results of Chou et al. The bacterial richness and gut microbiota diversity significantly increased at 49 days after HI (at the age of 13–15 weeks) in mice with either acute or chronic HBV infection. This indicated the increasing commensal bacteria that colonized the gut of mice appeared to be delayed by HBV infection. Several factors can influence commensal bacterial colonization, including the host immune system (Macpherson and Ganal-Vonarburg, 2018). Immunoglobulin A (IgA) is the main antibody isotype in the gut, which can coat live commensal bacteria and promote their adherence to the intestinal epithelial cells. Several studies have shown that IgA can affect the composition of the gut microbiota. In IgA-deficient mice, the inter-individual variability of the gut microbiota increased (Kubinak et al., 2015), while the diversity decreased (Fransen et al., 2015). Several types of commensal bacteria can modulate their surface architecture for them to be recognized by IgA, which facilitates bacterial colonization in the gut and helps exclude exogenous competitors (Donaldson et al., 2018). Increasing serum IgA has been found in patients with chronic liver diseases and has been identified as a potential biomarker of cirrhosis in patients with chronic hepatitis B (Lin et al., 2016). In this study, we also found that the concentration of IgA in the sera was significantly higher in the early phase in mice with chronic infection (data not shown). Therefore, IgA may play a role in commensal bacterial colonization in the gut during chronic HBV infection, which needs further studies.

The most common organisms in the mouse gut microbiota are members of the phyla *Bacteroidetes* and *Firmicutes*. In our study, the abundance of *Bacteroidetes* decreased and that of *Firmicutes* increased by day 14 in mice with either acute or chronic HBV infection, indicating that the *Firmicutes*/*Bacteroidetes* ratio increased after HBV infection. The alteration in this ratio is a marker or risk factor for several diseases, including alcoholic liver disease (Shao et al., 2018), primary Sjögren's syndrome, systemic lupus erythematosus (van der Meulen et al., 2019), obesity (Koliada et al., 2017), and aging (Lee et al., 2018). Bacteroidetes is critical for short-chain fatty acid production in the host (Wexler and Goodman, 2017). Butyrate, a short-chain fatty acid, can induce extrathymic Treg differentiation (Arpaia et al., 2013; Furusawa et al., 2013). *Firmicutes* can absorb and consume more calories than Bacteroidetes (Turnbaugh et al., 2006). HBV-specific immune response requires increasing energy consumption (Fisicaro et al., 2017). Therefore, the alteration in the *Firmicutes*/*Bacteroidetes* ratio may help shape the HBV-specific immune response via fatty acid metabolism and/or energy consumption, which needs further investigation. The *Firmicutes*/*Bacteroidetes* ratio decreased by day 49 after HBV infection, perhaps due to the adaptive mechanisms of the gut mucosal response to restore homeostasis (Assas et al., 2014; Shao et al., 2018). *Akkermansia* was dynamically changed in the control mice yet unchanged in mice with HBV infection. *Akkermansia* has been reported to play a protective role in alcoholic liver disease (Grander et al., 2018) and promote PD-1-based immunotherapy against epithelial tumors (Routy et al., 2018).

The composition of gut microbiota is associated with hepatic function in patients with chronic hepatitis B (Wang et al., 2017). The dynamic changing pattern in the abundance of *Lactobacillu*s and *Bifidobacterium* was found to differ in mice with acute vs. chronic HBV infection. *Lactobacillus* and *Bifidobacterium* have beneficial effects on immune homeostasis and gut health, with roles in reducing allergic sensitization, modulating the immune response to bacterial infection, and protecting the intestinal barrier function (Ludwig et al., 2018). It needs to be confirmed whether the observed alterations in the gut microbiome contributed to the immune response after HBV infection.

Numerous studies have confirmed that the frequency of viral-specific T cells expressing IFN-γ is a critical factor determining the outcome of HBV infection (Bertoletti and Ferrari, 2003, 2012). Consistently, a higher frequency of viral-specific CD8^+^ T cells expressing IFN-γ in the liver was detected in mice with acute HBV infection, while this was not detected in the control mice and chronic HBV-infected mice. Furthermore, enhanced IFN-γ production in the colon was also found only in mice with acute HBV infection. The intestinal microbiota composition was shown to be altered in mice with influenza infection, which was mediated by IFN-γ produced by lung-derived CCR9^+^CD4^+^ T cells recruited into the small intestine (Wang et al., 2014a). Therefore, liver-derived IFN-γ-producing lymphocytes may also be responsible for the differential profiles of gut microbiota in acute and chronic HBV infections, which needs further investigation.

Overall, we found that HBV infection hampers the development of the gut microbiota community and dynamically changes the gut *Firmicutes*/*Bacteroidetes* ratio in the mouse model mimicking acute or chronic HBV infection. Interestingly, the dynamic changes in *Lactobacillu*s and *Bifidobacterium* differed between acute and chronic HBV infections; these findings may be related to the IFN-γ-mediated immune response. These data improve our understanding of the relationship between gut microbiota and HBV infection; further investigation is needed to clarify the mechanisms involved.

## Data Availability Statement

Publicly available datasets were analyzed in this study. This data can be found here: https://trace.ncbi.nlm.nih.gov/Traces/sra_sub/sub.cgi?login=pda.

## Ethics Statement

This study was carried out in accordance with the Guidelines of the National Institutes of Health for Animal Care and Use. The protocol was approved by the Institutional Animal Care and Use Committee at Tongji Medical College, Huazhong University of Science and Technology (Permit Number: S814).

## Author Contributions

QZ, DY, and JW designed the study. QZ and PX performed the animal experiments. XZho, XL, WG, and BZ collected fecal and serum samples. QZ, XZhe, BW, DY, and JW analyzed the data and wrote the paper.

### Conflict of Interest

The authors declare that the research was conducted in the absence of any commercial or financial relationships that could be construed as a potential conflict of interest.
