# Corrigendum: Hepatitis B Virus Infection Alters Gut Microbiota Composition in Mice

**DOI:** 10.3389/fcimb.2020.00490

**Published:** 2020-09-15

**Authors:** Qingfeng Zhu, Panpan Xia, Xin Zhou, Xiaoran Li, Weina Guo, Bin Zhu, Xin Zheng, Baoju Wang, Dongliang Yang, Junzhong Wang

**Affiliations:** Department of Infectious Diseases, Union Hospital, Tongji Medical College, Huazhong University of Science and Technology, Wuhan, China

**Keywords:** HBV infection, HBV mouse model, gut microbiota, microbiota composition, dynamic changes, immune response

In the original article, there was an error in the Funding statement. The correct grant numbers for National Natural Science Foundation of China is 81501748, 91642118, 81461130019.

In the original article, there was an error in affiliation **1**. Instead of “Department of Infectious Diseases, Tongji Medical College, Union Hospital, Huazhong University of Science and Technology, Wuhan, China,” it should be “Department of Infectious Diseases, Union Hospital, Tongji Medical College, Huazhong University of Science and Technology, Wuhan, China”.

The authors apologize for these errors and state that this does not change the scientific conclusions of the article in any way. The original article has been updated.

