# Supplementary material for: Hepatitis B Virus Infection Alters Gut Microbiota Composition in Mice
Source: Front Cell Infect Microbiol. 2019 Nov 5;9:377. doi: 10.3389/fcimb.2019.00377 (PMC6848375; doi:10.3389/fcimb.2019.00377)
Supplement: Supplementary file 1 [file Data_Sheet_1.pdf]

Supplementary Material

Bioinformatics analysis

In this study, the OTU table was normalized by rarefaction to an even sequencing depth in order to remove sample heterogeneity. The rarefied OTU table was used to calculate alpha diversity indices including the OTU count and Shannon–Weaver index.

Illumina MiSeq sequencing yielded pair-end sequence data. First, the pair-end reads were merged into a sequence according to their overlap relationship, and then the result of the merge was evaluated. Furthermore, the quality filter of reads was conducted. The samples were distinguished based on the barcodes and primer sequences at both ends of the sequence and obtained valid sequences. After correcting the direction of the sequence, optimized data were obtained.

Data decontamination methods and parameters:

(1) The 300-bp reads were truncated at any site receiving an average quality score of < 20 over a 50-bp sliding window, discarding the truncated reads that were shorter than 50 bp. Reads containing N bases were also removed.

(2) The pair-end reads were merged into a sequence according to their overlap relationship, and the minimum overlap length was 10 bp.

(3) The maximum mismatch ratio allowed in the overlap area of the merged sequence was 0.2.

(4) The samples were distinguished and the direction of sequence was corrected based on the barcode and primer sequences at both ends of the sequence; the number of mismatches allowed in the barcode was 0 and the maximum number of mismatched primers was 2.

Software: FLASH and Trimmomatic

OTU clustering process:

(1) In order to reduce the amount of redundant calculation in the process of analysis, non-repetitive sequences were obtained from optimized data (http://drive5.com/usearch/manual/dereplication.html).

(2) Singletons were discarded (http://drive5.com/usearch/manual/singletons.html).

(3) With 97% clustering, an OTU sequence should be at least 3% different from all other OTUs, and OTU representative sequence should be the most abundant sequences in its neighborhood. During the clustering process, chimeric sequences were discarded.

(4) All optimized sequences to OTU representation sequences were mapped, and sequences with a similarity of > 97% were selected to generate an OTU table.

1.1 Supplementary Figures

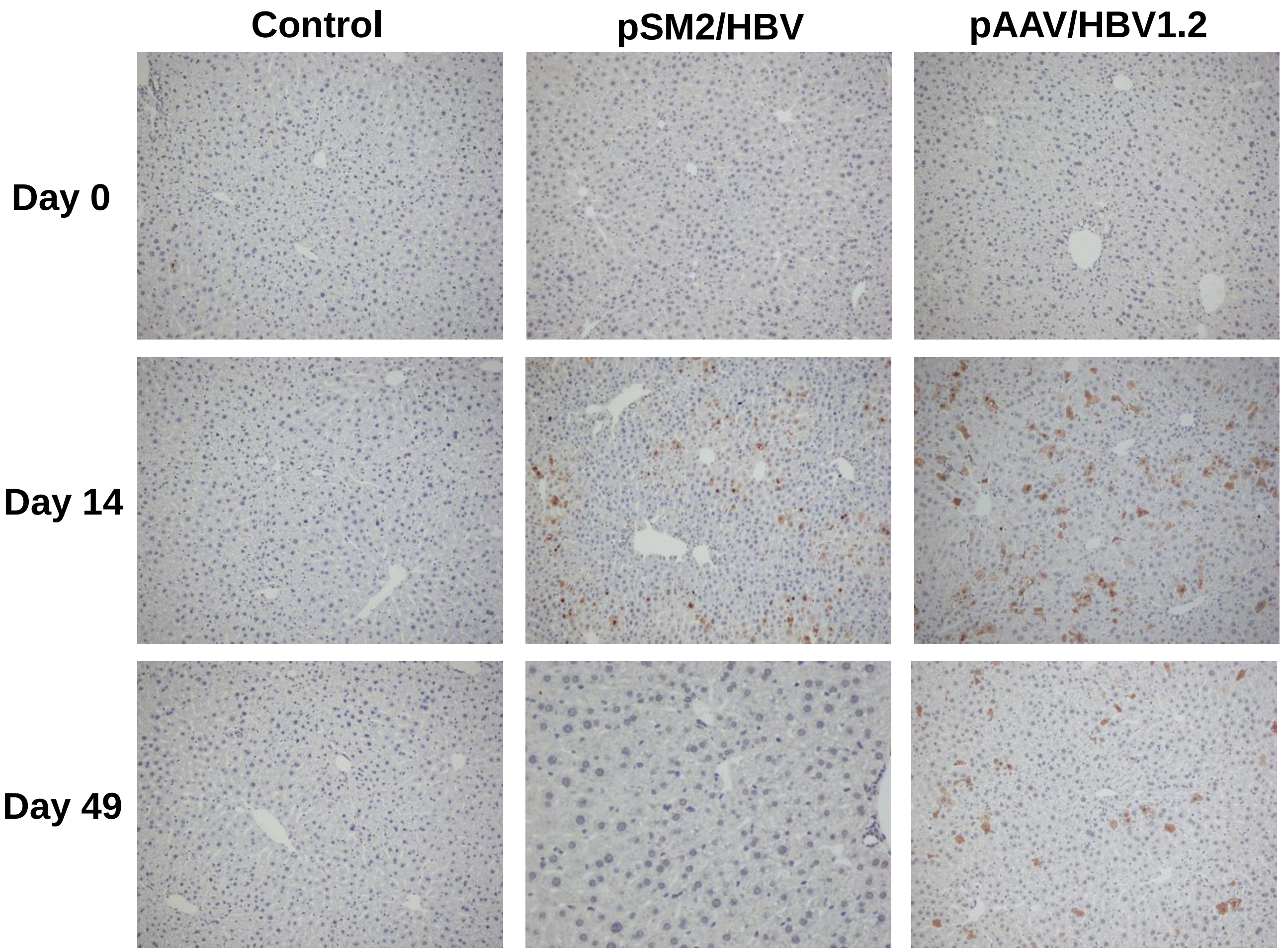

Supplementary Figure S1. HBcAg expression in the liver tissue.

The liver samples were collected from the control, pSM2/HBV, and pAAV/HBV1.2 mice, and intrahepatic HBcAg was detected by immunohistochemistry (magnification 200×)

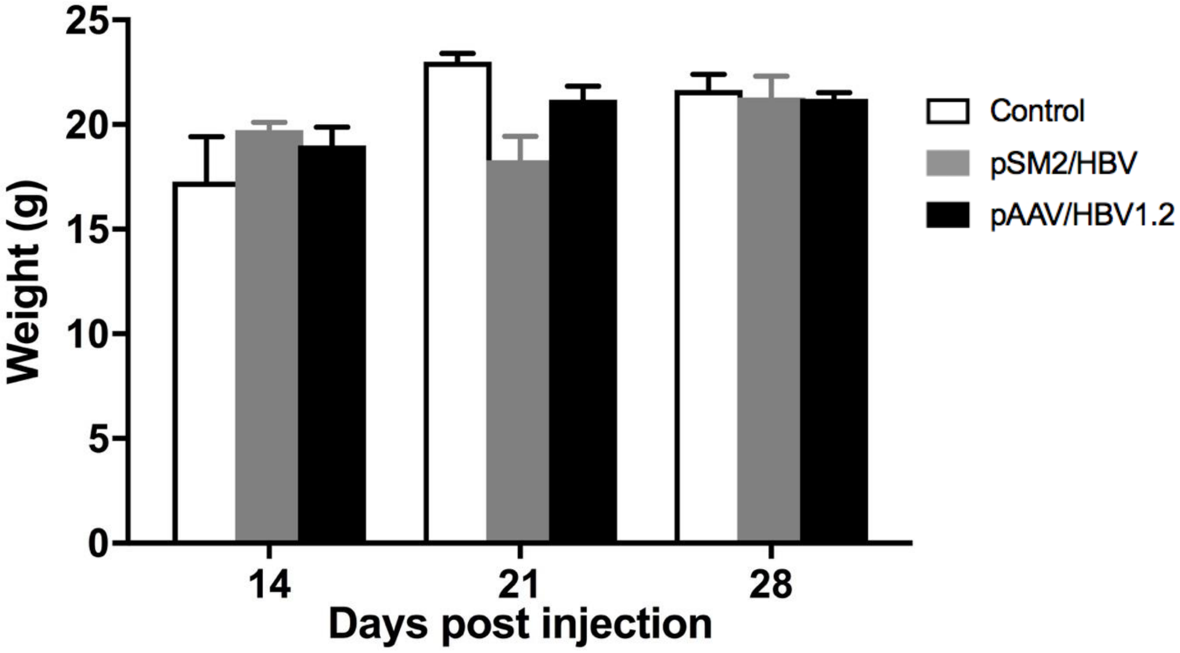

Supplementary Figure S2. Dynamic changes in mouse body weight

The body weight of the mice as monitored at different time points. Control mice, n = 10; pSM2/HBV HI mice, n = 9; pAAV/HBV1.2 HI mice, n = 9.

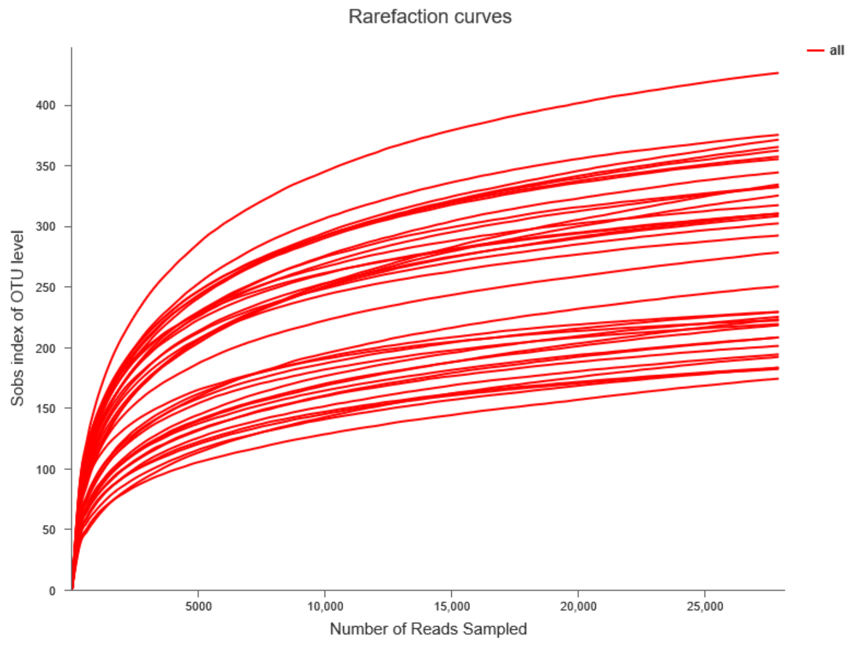

Supplementary Figure S3. Rarefaction curves of bacterial richness of each sample of gut microbiota in mice.

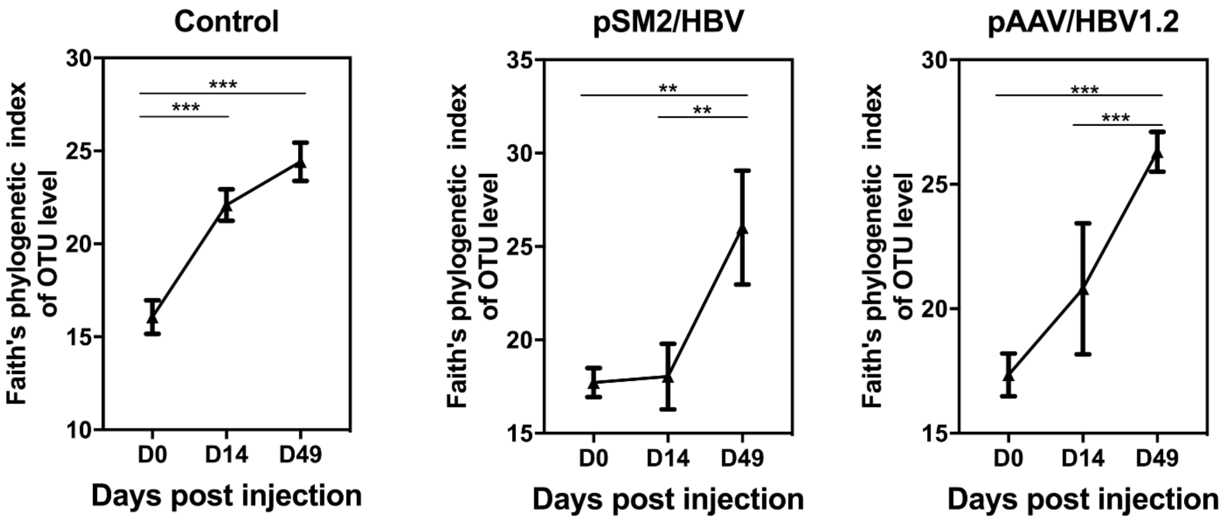

Supplementary Figure S4. Dynamic changes in Faith's phylogenetic diversity of gut microbiota in the control, pSM2/HBV HI, and pAAV/HBV1.2 HI mice

Faith's phylogenetic diversity of the gut microbiota at different time points in the control, pSM2/HBV HI, and pAAV/HBV1.2 HI mice is shown. Control mice, n = 3; pSM2/HBV HI mice, n = 3; pAAV/HBV1.2 HI mice, n = 3–7. *$p < 0.05$, *** $p < 0.01$, *** $p < 0.001$.

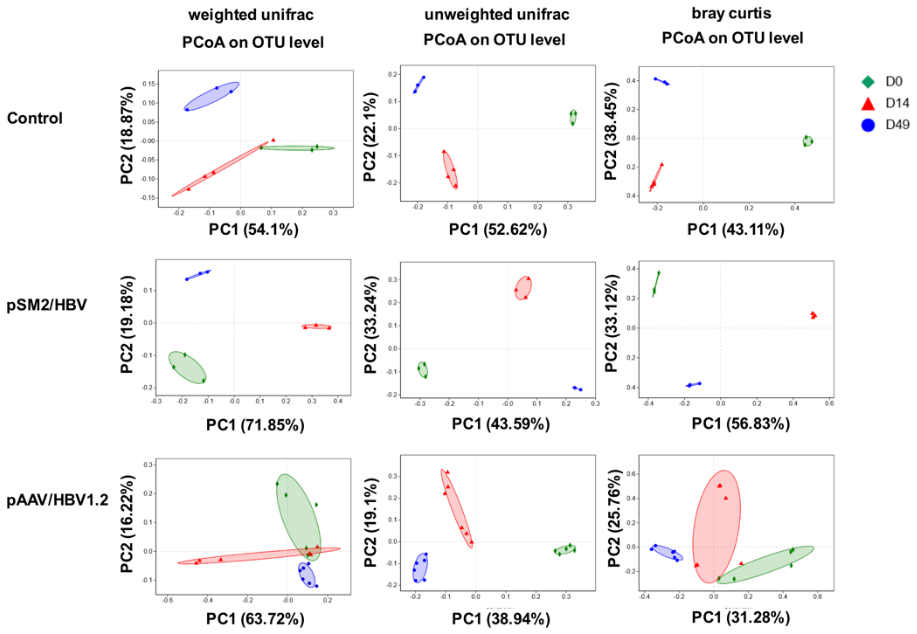

Supplementary Figure S5. Beta diversity of the gut microbiota in mice was analyzed using PCoA of weighed unifrac, unweighted unifrac, and Bray–Curtis distance matrix. n = 3–7

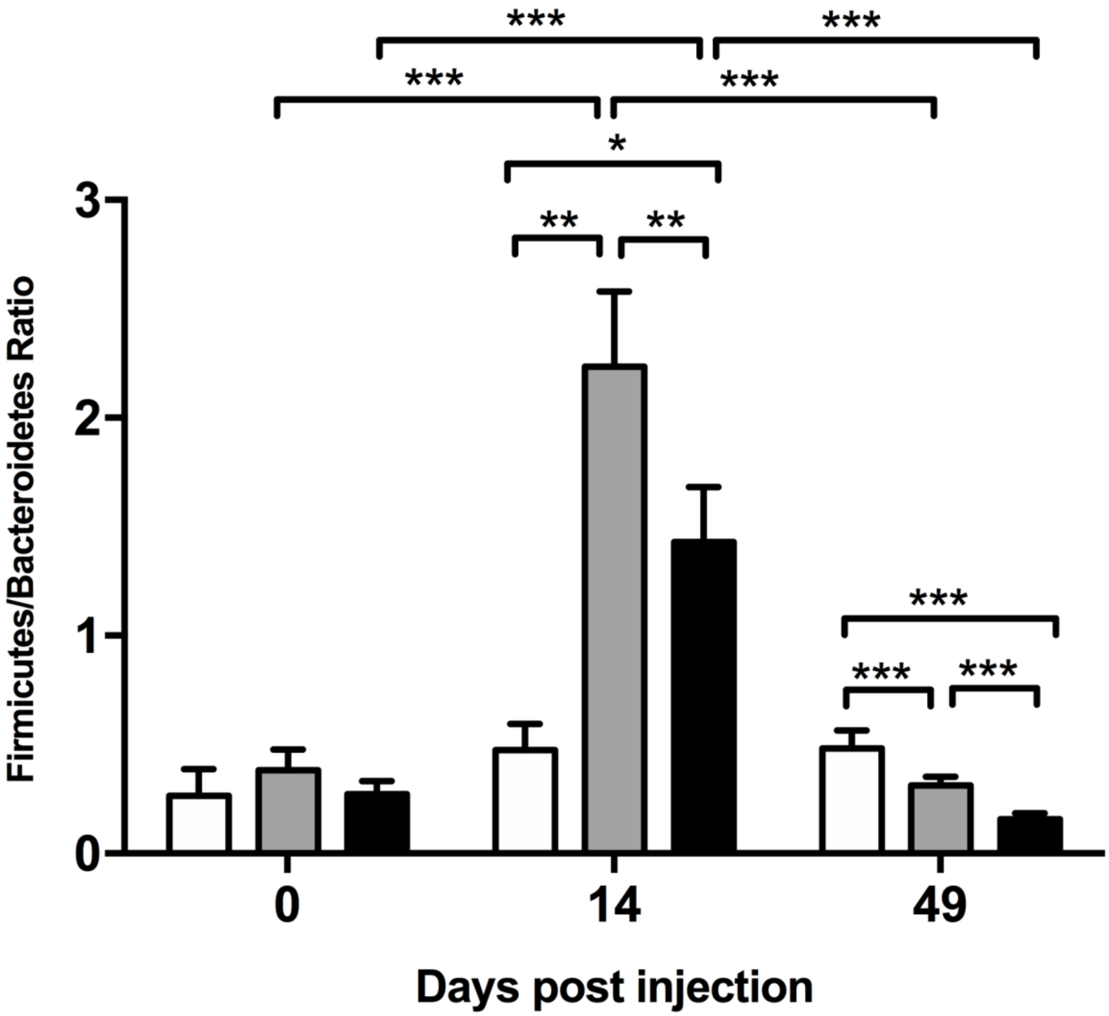

Supplementary Figure S6. Changes in the *Firmicutes*/*Bacteroides* ratio in the gut microbiota in the control, pSM2/HBV HI, and pAAV/HBV1.2 HI mice

The ratio of *Firmicutes*/*Bacteroides* was calculated with the relative abundance of Bacteroidetes and Firmicutes in the control, pSM2/HBV HI, and pAAV/HBV1.2 HI mice at different time points. Control mice, n = 3; pSM2/HBV HI mice, n = 3; pAAV/HBV1.2 HI mice, n = 3-7. *p < 0.05, *** p < 0.01, *** p < 0.001.
